# Nomogram for preoperative estimation of histologic grade in gastrointestinal neuroendocrine tumors

**DOI:** 10.3389/fendo.2022.991773

**Published:** 2022-10-24

**Authors:** Zhi-Qi Wu, Yan Li, Na-Na Sun, Qin Xu, Jing Zhou, Kan-Kan Su, Hemant Goyal, Hua-Guo Xu

**Affiliations:** ^1^ Department of Laboratory Medicine, The First Affiliated Hospital of Nanjing Medical University, Nanjing, Jiangsu, China; ^2^ Branch of National Clinical Research Center for Laboratory Medicine, Nanjing, Jiangsu, China; ^3^ Academy for Advanced Interdisciplinary Studies, Peking University, Peking, China; ^4^ Department of Radiology, the First Affiliated Hospital of Nanjing Medical University, Nanjing, Jiangsu, China; ^5^ Department of Laboratory Medicine, Jurong Hospital Affiliated to Jiangsu University, Jurong, China; ^6^ Department of Internal Medicine, Mercer University School of Medicine, Macon, GA, United States

**Keywords:** GI-NETs, grade, preoperative estimation, serum biomarker, nomogram

## Abstract

**Background:**

The treatment strategies and prognosis for gastroenteropancreatic neuroendocrine tumors were associated with tumor grade. Preoperative predictive grading could be of great benefit in the selection of treatment options for patients. However, there is still a lack of effective non-invasive strategies to detect gastrointestinal neuroendocrine tumors (GI-NETs) grading preoperatively.

**Methods:**

The data on 147 consecutive GI-NETs patients was retrospectively collected from January 1, 2012, to December 31, 2019. Logistic regression was used to construct a predictive model of gastrointestinal neuroendocrine tumor grading using preoperative laboratory and imaging parameters.The validity of the model was assessed by area under the receiver operating characteristic curve (AUC), calibration curve, and decision curve analysis (DCA).

**Results:**

The factors associated with GI-NETs grading were age, tumor size, lymph nodes, neuron-specific enolase (NSE), hemoglobin (HGB) and sex, and two models were constructed by logistic regression for prediction. Combining these 6 factors, the nomogram was constructed for model 1 to distinguish between G3 and G1/2, achieving a good AUC of 0.921 (95% CI: 0.884-0.965), and the sensitivity, specificity, accuracy were 0.9167, 0.8256, 0.8630, respectively. The model 2 was to distinguish between G1 and G2/3, and the variables were age, tumor size, lymph nodes, NSE, with an AUC of 0.847 (95% CI: 0.799-0.915), and the sensitivity, specificity, accuracy were 0.7882, 0.8710, 0.8231, respectively. Two online web servers were established on the basis of the proposed nomogram to facilitate clinical use. Both models showed an excellent calibration curve through 1000 times bootstrapped dataset and the clinical usefulness were confirmed using decision curve analysis.

**Conclusion:**

The model served as a valuable non-invasive tool for differentiating between different grades of GI-NETs, personalizing the calculation which can lead to a rational treatment choice.

## Introduction

Neuroendocrine tumors (NETs) are heterogeneous tumors arising in the secretory cells of the diffuse neuroendocrine system. About two-thirds of NETs which originate in the gastrointestinal system or pancreas (1, 2). Over the past few decades, the incidence of gastroenteropancreatic neuroendocrine tumors is increasing (3, 4), among which gastrointestinal neuroendocrine tumors (GI-NETs) have been the second most common GI malignant tumor (5, 6). Surgery resection is frequent options treatment modality for gastroenteropancreatic neuroendocrine tumors even with limited metastatic, which allows very high rates of definitive cure (7). A United States National Cancer Database study on 14,510 patients with gastroenteropancreatic neuroendocrine tumors revealed that resection of primary tumor and Grade 1 and 2 tumors was associated with prolonged survival (p< 0.001) (8). For small tumors and certain selected patients with metastatic disease, surgery remains the only curative modality (9–11). However, given the natural history and prognosis, the balance between the benefits and risks of surgery should be carefully evaluated (7). Treatment options for advanced gastroenteropancreatic neuroendocrine tumors include surgical, medical, interventional radiology and nuclear medicine strategies (12).

Grading of GI-NETs by histopathology is based on postoperative specimens.To facilitate the management of gastroenteropancreatic neuroendocrine tumors, the World Health Organization (WHO) has developed a classification system that can grade tumors based on the markers of cell proliferation in the biopsy specimens (13). An accurate gastroenteropancreatic neuroendocrine tumors graded preoperative evaluation can help choose the appropriate surgical modality to assess the patient’s risk-benefit profile. When gastroenteropancreatic neuroendocrine tumors are graded as G1 or G2, surgical/endoscopic resection may be the preferred treatment of choice (14). Therefore, it is important to grade the patient preoperatively to help make individualized treatment decisions.

Superficial GI-NETs with small tumor size are usually treated by endoscopic removal by mucosal resection (EMR) or submucosal dissection (ESD), which has demonstrated its advantages in treatment (15–18). The outcomes and prognosis often depend on the endoscopists’ clinical experience while monitoring for tumor metastasis or recurrence after surgery (19–21). However, There is an increased risk of tumor recurrence in patients with resected G2 or G3 GI-NETs with high risk features (size >2 cm, or positive lymph nodes) (21, 22).

Imaging helps confirm the location of the primary gastroenteropancreatic neuroendocrine tumors and also determines the presence of metastatic disease. In a retrospective study on 138 pancreatic NETs (pNETs) patients (104 in training and 34 in validation cohorts) with fusion radiological features and tumor margins, Gu et al. developed a radiomics nomogram to differentiate between G1 and G2/3 grades. The nomogram showed a strong discrimination with AUC of 0.974 (95%CI: 0.950-0.998) in the training and 0.902 (95%CI:0.798-1.000) in the validation cohort with good calibration (23). Feng et al. found that CT can be useful in the differentiaon of G1/2 grades to G3 gastroenteric NETs (24). However, due to the difficulty of data extraction and computational complexity, both radiologists and clinicians should be familiar with the indications and interpretations of imaging modalitie (25). Therefore, it is necessary to construct models that could be graded by a combination of routine imaging and routine blood tests for clinical convenience use. Serum biomarkers are being widely used in the diagnosis, follow-up and prognosis of malignanies because they are non-invasive, easy to obtain, and economical. However, gastroenteropancreatic neuroendocrine tumors are highly heterogeneous tumors, and the role of a single serum biomarker is still unclear (26, 27). Therefore, we have combined the imaging and serum biomarker characteristics of GI-NETs to develop two nomogram models for preoperative estimation of the histological grades.

## Methods

### Material and methods

This analysis was reported according to the TRIPOD (Transparent reporting of a multivariable prediction model for individual prognosis or diagnosis) guidelines (28).

### Study design and participants

The imaging and laboratory data of the 147 consecutively diagnosed GI-NETs patients from January 1, 2012, and December 31, 2019, were retrospectively collected from the First Affiliated Hospital of Nanjing Medical University. This study protocol conforms to the ethical guidelines of the 1975 Declaration of Helsinki and approved by the Ethics Committee of The First Affiliated Hospital of Nanjing Medical University (Nanjing, China) (Ethical approval No. 2020-SR- 012).

The inclusion criteria were: (1) histopathologically confirmed GI-NETs; (2) documentation of histologic grade (G1/2/3); (3) availbility of complete imaging, laboratory data and endoscopy results. Patients who had incomplete clinical data, underwent treatments before the consultation or had unsatisfactory test samples were excluded. All clinical data were used to develop the models.

### Clinical characteristics of the patients

All potential predictors were selected based on the detailed literature reviews and published clinical evidence. All risk factors previously reported to contribute to a higher grade of GI-NETs were used within the confines of data availability. The data on routine diagnostic tests, including laboratory and imaging tests, were extracted from the medical records of each patient.

The collected clinical variables are reported in **Table 1**. The serum biomarkers included hemoglobin (HGB), albumin (ALB), lactate dehydrogenase (LDH), and the tumor marker sets, including alpha-fetoprotein (AFP), carcinoembryonic antigen (CEA), cancer antigen 19-9 (CA19-9), cancer antigen 72-4 (CA72-4), cytokeratin 19 fragment 21-1 (Cyfra21-1) and neuron-specific enolase (NSE). The imaging features of the tumor included mucosa (normal or abnormal), boundary (clear or unclear), ulceration (absent or present), and morphology (regular or irregular), areas of growth pattern (node/knub or no-node/knub), lymph node (no or yes), degree of enhancement (unmarked or marked), and distant organ metastases (no or yes), and the tumor size(<2 or ≥2). Distant organ metastasis was defined as metastases found in organs other than the primary lesion (e.g., liver, lungs, and bone). All preoperative imaging data was carried out independently by two experienced radiologists.

**Table 1:** Characteristics of patients with GI-NETs in the G1, G2 and 3 groups.

	G1 (n = 62)	G2 (n = 24)	G3 (n = 61)
Age, median (IQR), y	53.5 (47.0-62.0)	54.5 (49.8-63.3)	63.0 (58.0-70.0)
AFP, median (IQR), ng/mL	2.4 (1.8-3.2)	2.3 (1.8-3.3)	2.6 (1.8-3.5)
CEA, median (IQR), ng/mL	1.5 (1.1-2.3)	1.9 (1.1-3.0)	2.4 (1.8-5.2)
CA199, median (IQR), U/L	9.0 (6.0-13.6)	9.4 (7.4-14.8)	9.3 (5.6-19.0)
CA724, median (IQR), U/L	1.7 (1.0-2.9)	1.2 (0.9-3.8)	1.4 (1.0-2.6)
Cyfra21-1, median (IQR), ng/mL	1.7 (1.2-2.3)	1.4 (1.2-2.3)	2.0 (1.5-3.3)
NSE, median (IQR), ng/mL	15.0 (13.1-18.3)	16.2 (14.8-20.8)	21.1 (16.0-28.7)
ALB, median (IQR), g/L	40.7 (37.8-43.6)	40.4 (37.6-42.1)	37.9 (35.7-41.2)
HGB, median (IQR), g/L	133.5 (123.2-144.2)	139.0 (116.2-146.5)	128.0 (115.0-139.0)
LDH, median (IQR), U/L	181.0 (169.0-207.5)	189.0 (161.0-203.0)	190.5 (162.0-246.5)
Sex, Female, No. (%)	33 (53.2)	11 (45.8)	17 (27.9)
Mucosa, Abnormal (%)	11 (17.7)	8.0 (33.3)	43 (70.5)
Boundary, Unclear (%)	54 (52.9%)	13 (28.3)	41 (73.2)
Ulceration, Present (%)	3.0 (4.8)	5.0 (20.8)	28 (45.9)
Morphology, Irregular (%)	35 (56.5)	13 (54.2)	52 (85.2)
Growth Pattern, Knub (%)	26 (41.9)	12 (50.0)	12 (19.7)
Lymph node, YES (%)	2.0 (3.2)	5.0 (20.8)	37 (60.7)
Degree of Enhancement, YES (%)	32 (51.6)	20 (83.3)	48 (80.0)
Distant Organ Metastasis, YES (%)	0.0 (0.0)	3.0 (12.5)	10 (16.4)
Tumor size ≥2, No. (%)	16 (25.8)	12 (50.0)	52 (85.3)

AFP, alpha-fetoprotein; CEA, carcinoembryonic antigen; CA19-9, carbohydrate antigen 19-9; CA72-4, cancer antigen 72-4; Cyfra21-1, cytokeratin 19 fragment 21-1; NSE, neuron-specific enolase; ALB, albumin; HGB, hemoglobin; LDH, lactate dehydrogenase; IQR, Inter Quartile Range.

The histologic grade was an immunohistochemical analysis of postoperative specimens according to 2010 WHO standards for NETs. Tumor grade was defined numerically, in which low-grade (G1) tumors had a mitotic rate from 0 to 1 per 10 high power fields (HPF) or a Ki-67 index from 0% to 2%, intermediate-grade (G2) tumors have a mitotic rate from 2 to 20 per 10 HPF or a Ki-67 index from 3% to 20%, and high grade (G3) tumors have a mitotic rate greater than 20 per 10 HPF or a Ki-67 index greater than 20% (1). Two experienced pathologists independently evaluated all specimens. The mutual discussion settled any controversies in the findings between the pathologists, and a final standard histopathological report on each patient was generated.

### Statistical analysis

Unpaired two-tailed t-tests or Mann-Whitney tests were used for continuous variables. Categorical variables were compared using the χ2 test or Fisher’s exact test. All patients with GI-NETs were included for variable selection and the model development. The significance of each variable in the dataset was assessed by logistic regression analysis to investigate the independent risk factors of the presence of G3 or G1. With 5-6 variables potentially associated with G1 or G3 with GI-NETs the minimum sample size required approximately 50-60 G1 or G3 to avoid violating the principle of approximately ten outcome events per variable in the regression (29).

A nomogram was developed based on the results of multivariate logistic regression analysis. Based on the nomogram of tumor pathologic grade classifier determined, an online Calculator automatically calculating the individual risk of the pathologic grade was also generated. An internal validation procedure was performed using bootstrap method to estimate the potential performance of the model based on calibration and identification, which was chosen to optimize the number of available cases while minimizing the risk of overfitting (30). A decision curve analysis was performed to assess the clinical usefulness and the net benefits of nomogram-assisted decision.

The Youden Index was used to determine the best threshold in the ROC analysis. Accuracy of the optimal cutoff value was assessed by the sensitivity, specificity, predictive values, and likelihood ratios. All analyses were performed using R, version 3.6.1 (http://www.r-project.org/). In all analyses, P<0.05 was considered to indicate statistical significance.

## Results

### Patients’ characteristics

The patients’ data are presented as median and interquartile range for continuous variables and frequencies(percentages) for categorical variables. All variables used in this analysis were based on data obtained prior to treatment. The dataset consisted of 147 patients with GI-NETs, with a median age of 58 years and 86 (58.50%) male patients. The clinical characteristics of the patients with GI-NETs including clinical laboratory data and clinical imaging features are listed in **Table 1**. In the dataset, histopathologically identified G1, G2, and G3 GI-NETs were found in 62, 24, and 61 patients, respectively.

### Development and validation of a nomogram to differentiate G3 from G1/2 in GI-NETs

Considering the potential impact of each patient’s clinical characteristics, univariate and multivariate logistic regression analyses were applied (**Table 2**). The multivariate analysis of the final model was reported as an odds ratio (OR, 95%CI). The tumor size (2.56 [1.53-4.56]), lymph node (4.06 [1.28-13.96]), serum NSE (3.98 [1.18-18.30]), HGB (0.96[0.90-0.99]), age (1.10 [1.05-1.17]) and sex (3.51 [1.10-12.10]) were independently associated with G3 grade (**Table 3**). These independently associated risk factors were used to form a G3 and G1/2 estimation model. The nomogram and online risk calculator were built based on the diagnostic model. The nomogram was based on the conversion of each regression coefficient in multiple logistic regression to a ratio of 0 to 100 points. The effect of the variable with the highest β coefficient (absolute value) was assigned 100 points. The points were added to the independent variables to get the total points, which were converted to predicted probabilities. The online risk calculator used each parameter to personalize the calculation of the probability of being G3 for the patient.

**Table 2:** Univariate and Multifactor Logistic Regression Model for Predicting in 147 Patients.

Characteristic	G1 vs G2/3		G3 vs G1/2	
	univariate regression	Multifactor regression	univariate regression	Multifactor regression
Sex, male vs female	0.014	0.152	0.005	0.063
Age, y	0.002	0.047	0.002	0.001
AFP, ng /ml	0.315		0.102	
CEA, ng /ml	0.107		0.039	0.564
CA19-9, U/ml	0.355		0.277	
CA72-4, U/ml	0.364		0.399	
CYFRA21-1, ng /ml	0.046	0.950	0.005	0.459
LDH, U/L	0.074		0.017	0.678
NSE, ng /ml	0.001	0.066	0.002	0.049
HGB, g/L	0.017	0.366	0.004	0.063
ALB, g/L	0.003	0.569	0.001	0.260
Growth Pattern (No-Node vs Knub)	0.085		0.003	0.944
Boundary (Unclear vs Clear)	0.001	0.115	0.001	0.200
Morphology (Regular vs Irregular)	0.001	0.637	0.001	0.436
Mucosa (Abnormal vs Normal)	0.001	0.926	0.001	0.481
Ulceration (Present vs Absent)	0.001	0.138	0.001	0.394
Lymph node (Yes vs No)	0.001	0.006	0.005	0.004
Degree of Enhancement (Yes vs No)	0.001	0.388	0.001	0.314
organ metastases (Yes vs No)	0.987		0.013	0.706
Tumor size (≥2 vs <2 )	0.009	0.012	0.004	0.001

AFP, alpha-fetoprotein; CEA, carcinoembryonic antigen; CA19-9, carbohydrate antigen 19-9; CA72-4, cancer antigen 72-4; Cyfra21-1, cytokeratin 19 fragment 21-1; NSE, neuron-specific enolase; HGB, hemoglobin; ALB, albumin; LDH, lactate dehydrogenase.

**Table 3:** Multivariable Logistic Regression Model for Predicting G3 vs G1/2 and G1 vs G2/3.

Characteristics	G1 vs G2/3		G3 vs G1/2	
	OR (95%CI)	P Value	OR (95%CI)	P Value
sex			3.51 (1.10, 12.10)	0.036
HGB			0.96 (0.90, 0.99)	0.008
age	1.04 (1.00, 1.08)	0.049	1.10 (1.05, 1.17)	0.000
Ln NSE	3.98 (0.94 19.34)	0.066	3.98 (1.18, 18.30)	0.032
Lymph node	8.80 (2.24, 58.79)	0.004	4.06 (1.28, 13.96)	0.028
Tumor size	2.07 (1.38, 3.19)	0.001	2.56 (1.53, 4.56)	0.000

OR, odds ratio; CI, confidence interval; HGB, hemoglobin; Ln, natural logarithms transformed; NSE, neuron-specific enolase.

**Figure 1:**
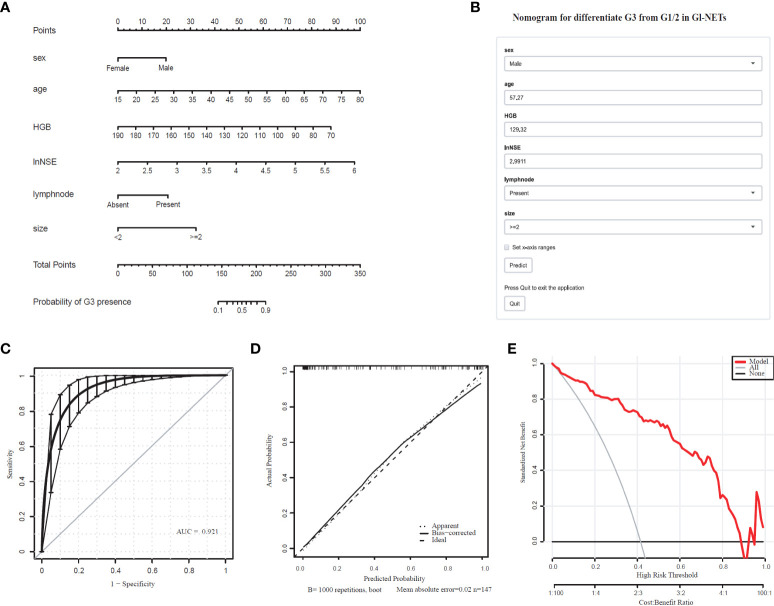
Nomogram for Preoperative Estimation of G3 Risk and Its Predictive Performance. **(A)**, Nomogram to estimate the risk of G3 presence in GI-NETs. **(B)**, corresponding online calculators to predict the risk of G3. Online tools are available at http://ginet.shiny.io/dynnomapp/. **(C)**, Receiver operating characteristic ROC. **(D)**, the calibration curve and **(E)**, the decision curve.

The differentiated nomogram of G3 and G1/2 in GI-NETs and an online risk calculator were constructed based on the full model (**Figures 1A, B**). Due to the wide range (9.7-370) ng/mL, the NSE values were converted to natural logarithms and written as lnNSE to mitigate the skewness and achieve a better fit. The AUC of the prediction model for G3 and G1/2 reached 0.921 (95% CI: 0.884-0.965) (**Figure 1C**). The calibration curves showed accuracy in the nomogram predicted pathological tumor grade G3 and G1/2 (**Figure 1D**). DCA was used to demonstrate the clinical decision utility of this nomogram. The area under the decision curve shows the clinical utility of the corresponding strategy. The decision curve showed that the nomogram to inform clinical decisions was better than the scenarios when all the patients received the treatment or when none of the patients were treated (**Figure 1E**). The red in DCA shows more area than the treat all or no treatment strategies.

### Development and validation of a nomogram to differentiate G1 from G2/3 in GI-NETs

**Figure 2:**
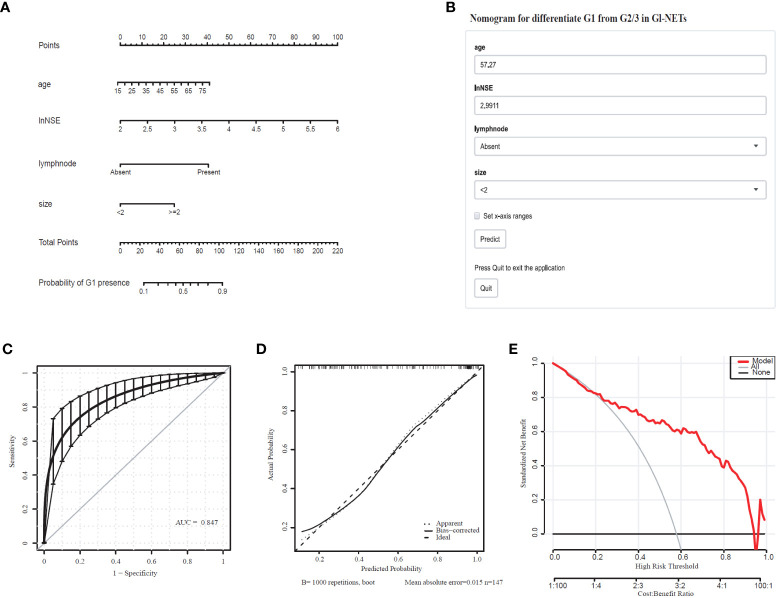
Nomogram for Preoperative Estimation of G1 Risk and Its Predictive Performance. **(A)**, Nomogram to estimate the risk of G1 presence in GI-NETs. **(B)**, corresponding online calculators to predict the risk of G1. Online tools are available at http://ginet.shiny.io/dynnomapp1/. **(C)**, Receiver operating characteristic ROC. **(D)**, the calibration curve and **(E)**, the decision curve.

Comprehensively considering the results in the model of univariate and multivariate logistic regression analyses, age, lymph node, and tumor size with significant difference (p < 0.05). Based on the previous studies, NSE (p=0.066) was included in the model as a variable. The tumor size (2.07 [1.38-3.19]), lymph node (8.80 [2.24-58.79]), lnNSE (3.98 [0.94-19.34]]), age (1.04 [1.00-1.08]), were independently associated with G1 (**Table 3**). These independently associated risk factors were used to form a G1 and G2/3 estimation nomogram. The differentiated nomogram of G1 and G2/3 in GI-NETs and an online risk calculator were constructed based on the full model (**Figures 2A, B**). The nomogram was used by adding up the points identified on the points scale for each variable, and the online risk calculator used each parameter to personalize the calculation of the probability of being G1 for the patient. The AUC of the prediction model for G1 and G2/3 reached 0.847 (95% CI: 0.799-0.915) (**Figure 2C**). The calibration curves and DCA showed accuracy in the nomogram predicted pathological tumor grade G1 and G2/3 (**Figures 2D, E**).

### Model setting and internal validation in the model development set

To validate the model performance of the nomogram, 1000 bootstrap calibrations were performedfor each of these two models using the dataset of 147 patients. The bootstrap-full scales showed the goodness of fit of the regression models. (**Figure 1D**) is for G3 and G1/2, (**Figure 2D**) for G1 and G2/3.

### Diagnostic value according to the nomogram cutoff for tumor pathologic grade classifier

The Youden index using optimal threshold maximization receiver operating characteristic curve analysis (ROC) was used to calculate the optimal cutoff value to assess the optimal threshold value predicting GI-NETs pathologic grade classifier of the model by sensitivity, specificity, and accuracy, as summarized in **Table 4**. This result demonstrated the high accuracy of the developed radiomics signature for the classification of G3 and G1/2 GI-NETs.

**Table 4:** Diagnostic performance of the model for grade status of GI-NET.

Method	G1 vs G2/3			G3 vs G1/2		
	SEN	SPE	ACC	SEN	SPE	ACC
sex				0.7213	0.5116	0.5986
HGB				0.9167	0.2791	0.5411
age	0.7529	0.5968	0.6886	0.8033	0.6395	0.7466
Ln NSE	0.6235	0.7419	0.7006	0.7049	0.7093	0.7153
Lymph node	0.4941	0.9677	0.6939	0.6066	0.9186	0.7891
Tumor size	0.7529	0.7419	0.7790	0.8525	0.6744	0.7483
Model	0.7882	0.8710	0.8231	0.9167	0.8256	0.8630

SEN, sensitivity; SPE, specificity; ACC, accuracy.

## Discussion

In recent years, nomograms are increasingly being used to construct the prediction models in the clinical practice because of its friendly visual graphical interface (31). Our study suggests that combining imaging and laboratory tests prior to endoscopy yielded a useful nomogram to predict tumor grade classification of GI-NETs. The model could stratify patients into G3 and G1/2 with an AUC of 0.921 (95% CI: 0.884-0.965), G1 and G2/3 with an AUC of 0.847 (95% CI: 0.799-0.915), which had good discrimination and goodness-of-fit.

Since the treatment strategies and prognosis for gastroenteropancreatic neuroendocrine tumors were associated with tumor grade, pre-treatment grading is valuable. Imaging studies on the grading of pNET showed that a combination of multiple imaging findings could distinguish G1 from G2/3 (32–34), and a multicenter study showed the same results (23). Liang and his colleagues developed a nomogram with the use of a combination of contrast-enhanced CT scan and clinical characteristics to differentiate the preoperative histologic grade of patients with pNETs, which well distinguished between G1 and G2/3 (35). Based on CT radiological scoring, tumor size in pNET patients were difference between grade 1 and grade 2, tending to be grade 1 when the size was less than 2 cm. Especially for pNET patients with tumor size less than 2 cm, the risk of G2/3 was low (36). This is consistent with our findings that patients with tumor size less than 2 cm are more likely to be in the G1 group.

The treatment strategies between neuroendocrine tumors (NET) and neuroendocrine carcinoma (NEC) are not the same, chemotherapy or radiotherapy is usually adopted for G3 tumors. It would be much more valuable in clinician selection of treatment modalities to show a model in the differentiation between G3 and G1/G2 (37). By using imaging features findings, grade 3 could also be differentiated from grade 1/2 (38–40). Using dynamic computed tomography, Horiguchi S et al. found that the proportion of the quantification value in the tumor to the pancreatic parenchyma in arterial phase could predict pathological grade 3 disease (41). However, the difficulty of data extraction and computational complexity requires experienced radiologists and limit the clinical application of imaging features in the prediction model of NET pathology grading (42). Therefore, we developed simple new models for easy clinical use, in which tumor size and lymph nodes were entered into the final model after performing logistic regression from 10 imaging features. The serum biomarkers added to this model could be well-differentiated G3 and G1/G2 with an AUC of 0.921 (95% CI: 0.884-0.965), sensitivities and specificities of 0.9167 and 0.8256, respectively.

Many serum biomarkers are available for the diagnosis of gastroenteropancreatic neuroendocrine tumors, but their specific diagnostic value is limited (26). NSE is mainly expressed in poorly differentiated tumors rather than in well-differentiated gastroenteropancreatic neuroendocrine tumors. Gut et al. found that NSE values were significantly higher among patients with NETs midgut type tumor with liver involvement. The progression of the disease was significantly higher in G2 than G1 group (p = 0.003) (43). In our study, the NSE could be directly entered into the G3 model. However, in the G1 model, NSE was put into the model based on the previous research experience (p =0.066) (44). NSE can be expressed in tumors other than NETs too. Mjønes et al. performed a retrospective study on 178 tumors (carcinomas and NETs) from the breast, lungs, stomach, and kidney and found that NSE was expressed in 78% of all the tumors. They found that NSE expression was positively associated with other additional neuroendocrine markers such as chromogranin A and synaptophysin. Moreover, it was concluded that the tumors of neuroendocrine origin have higher NSEstaining intensity and positive cells number (45). In another retrospective study on 592 sporadic stage IV gastroenteropancreatic neuroendocrine tumors, it was found that higher serum NSE level at the time of the first consultation was associated with a more aggressive disease course which could be used for prognostic use (46). A study of esophageal neuroendocrine carcinoma by Sohda M et al. found that patients with high serum NSE values had a significantly worse prognosis than those with normal NSE values and substantially fewer complete response cases in the high NSE group receiving chemotherapy (47). A study showed that the median NSE of G1, G2, and G3 non-functional p-NETs were 12.9, 14.9 and 16.5 μg/L respectively, showing statistical significance to differentiate different grades (48). Consistent with the results we found, NSE levels were significantly different between G1, G2 and G3, and it could be entered as a variable in the nomogram.The role of neuron-specific enolase may have been underestimated as a marker in NETs (45).

Studies on cancer have shown that low hemoglobin levels are associated with an increased risk of gastrointestinal bleeding, which has predictive value in patients with GI cancers, especially in advanced tumors (49–51). Our results showed that HGB appeared only in the G3 model with a high sensitivity of 0.9167 and a low specificity of 0.2791. And Hemoglobin levels have been shown to be directly linked to survival and tumor development in studies (52). Caro et al. showed that anemia is associated with shorter survival times for patients (53). This is consistent with our results. After performing stepwise regression, HGB was entered in models G3 and G1/2, but not in models G1 and G2/3. Therefore, the serum level of HGB can be a very useful clinical aid for evaluating the presence of malignant tumors at the time of diagnosis.

Based on the embryonic derivation, GI-NETs are classified from foregut (gastro-duodenal), midgut (jejunal, ileal, and cecal), and hindgut (distal colic and rectal) tumors (1). The clinical invasiveness of GI-NETs may vary depending on the primary site. The clinical and biological features of GI-NETs vary widely depending on its location, and the preferred surgical treatment varies depending on the site and size of the tumor (54). In contrast, the gastric and rectal NETs often have an indolent course, but they can progress rapidly once metastasized (1). Therefore, GI-NETs’ size thresholds recommended are different in different intestinalparts, according to the European Neuroendocrine Tumor Society (ENETS) (55). Here, we took the GI tract as a whole and tried to combine the results of imaging and laboratory tests in order to find a common method to perform the preliminary grading before endoscopy. Based on the two models for differentiating between grade 1 and grade 3 gastrointestinal neuroendocrine tumorscorrelate significantly with GI-NETs garde and provide a potentially valuable non-invasive tool for differentiating between different grades of GI-NETs. It provides physicians with a graded predictive tool prior to endoscopic treatment to select a personalized treatment strategy for patients with GI-NETs.

Some limitations of this study should be noted. First, possible data collection bias from retrospective studies might have occurred, and some patients were lost due to incomplete information. Secondly, this study is a single-center experience, and the results need to be validated in a multicenter study. Validation of big data could provide more basis for this study to eliminate this bias. In addition, a prospective study should be done to validate the role of NSE in GI-NETs.

## Conclusions

By retrospectively investigating the patients with GI-NETs in our datasets the results of imaging, serological data, sex, and age, we constructed two diagnostic models. The models could be used as preoperative tools for distinguishing grade 1/2 from 3 and grade 1 from 2/3 in patients with GI-NETs separately.

## Data availability statement

The original contributions presented in the study are included in the article/supplementary material. Further inquiries can be directed to the corresponding author.

## Ethics statement

The studies involving human participants were reviewed and approved by the Ethics Committee of The First Affiliated Hospital of Nanjing Medical University. Written informed consent for participation was not required for this study in accordance with the national legislation and the institutional requirements.

## Author contributions

H-GX designed the study. Z-QW, YL, N-NS, and QX contributed to the generation, collection, assembly, analysis and/or interpretation of data. JZ and K-KS performed the statistical analysis. Z-QW and YL wrote the manuscript. H-GX and HG revised the manuscript. All the authors have read and approved the final manuscript. All authors contributed to the article and approved the submitted version.

## Funding

This work was supported by the National Natural Science Foundation of China (81302531), Natural Science Foundation of Jiangsu Province of China (BK20181492), the National Key Clinical Department of Laboratory Medicine of China in Nanjing, Key laboratory for Laboratory Medicine of Jiangsu Province (ZDXKB2016005) and by the Priority Academic Program Development of Jiangsu Higher Education Institutions.

## Conflict of interest

The authors declare that the research was conducted in the absence of any commercial or financial relationships that could be construed as a potential conflict of interest.

## Publisher’s note

All claims expressed in this article are solely those of the authors and do not necessarily represent those of their affiliated organizations, or those of the publisher, the editors and the reviewers. Any product that may be evaluated in this article, or claim that may be made by its manufacturer, is not guaranteed or endorsed by the publisher.

## Abbreviations

GI-NETs, gastrointestinal neuroendocrine tumors; AUC, area under the receiver operating characteristic curve; ROC, receiver operating characteristic curve; DCA, decision curve analysis; IQR, inter quartile range; HGB, haemoglobin; ALB, albumin; LDH, lactate dehydrogenase; AFP, alpha-fetoprotein; CEA, carcinoembryonic antigen; CA19-9, cancer antigen 19-9; CA72-4, cancer antigen 72-4; Cyfra21-1, cytokeratin 19 fragment 21-1; NSE, neuron-specific enolase; HPF, high power fields; WHO, World Health Organization; EMR, endoscopic removal by mucosal resection; ESD, submucosal dissection; pNETs, pancreatic NETs; NET, Neuroendocrine tumors; NEC, neuroendocrine carcinoma; ENETS, European Neuroendocrine Tumor Society.

## References

[1] Cives M Strosberg JR. Gastroenteropancreatic neuroendocrine tumors. CA Cancer J Clin (2018) 68:471–87. doi: 10.3322/caac.21493 30295930

[2] Sigel CS. Advances in the cytologic diagnosis of gastroenteropancreatic neuroendocrine neoplasms. Cancer Cytopathol (2018) 126:980–91. doi: 10.1002/cncy.22073 30485690

[3] Tai WM Tan SH Tan DMY Tai WM Tan SH Tan DMY. Clinicopathologic characteristics and survival of patients with gastroenteropancreatic neuroendocrine neoplasm in a multi-ethnic Asian institution. Neuroendocrinology. (2019) 108:265–77. doi: 10.1159/000495140 30399612

[4] Das S Dasari A. Epidemiology, incidence, and prevalence of neuroendocrine neoplasms: Are there global differences? Curr Oncol Rep (2021) 23:43. doi: 10.1007/s11912-021-01029-7 33719003 PMC8118193

[5] Modlin IM Oberg K Chung DC Jensen RT de Herder WW Thakker RV. Gastroenteropancreatic neuroendocrine tumours. Lancet Oncol (2008) 9:61–72. doi: 10.1016/S1470-2045(07)70410-2 18177818

[6] Lee MR Harris C Baeg KJ Aronson A Wisnivesky JP Kim MK. Incidence trends of gastroenteropancreatic neuroendocrine tumors in the united states. Clin Gastroenterol Hepatol (2019) 17:2212–2217 e1. doi: 10.1016/j.cgh.2018.12.017 30580091

[7] Sauvanet A. Gastroenteropancreatic neuroendocrine tumors: Role of surgery. Ann Endocrinol (Paris) (2019) 80:175–81. doi: 10.1016/j.ando.2019.04.009 31079831

[8] Tierney JF Chivukula SV Wang X Pappas SG Schadde E Hertl M. Resection of primary tumor may prolong survival in metastatic gastroenteropancreatic neuroendocrine tumors. Surgery. (2019) 165:644–51. doi: 10.1016/j.surg.2018.09.006 30366604

[9] Berardi R Rinaldi S Torniai M Morgese F Partelli S Caramanti M. Gastrointestinal neuroendocrine tumors: Searching the optimal treatment strategy–a literature review. Crit Rev Oncol Hematol (2016) 98:264–74. doi: 10.1016/j.critrevonc.2015.11.003 26643525

[10] Singh S Chan DL Moody L Liu N Fischer HD Austin PC. Recurrence in resected gastroenteropancreatic neuroendocrine tumors. JAMA Oncol (2018) 4:583–5. doi: 10.1001/jamaoncol.2018.0024 PMC5885199 29543939

[11] Zaidi MY Lopez-Aguiar AG Dillhoff M Beal E Poultsides G Makris E. Prognostic role of lymph node positivity and number of lymph nodes needed for accurately staging small-bowel neuroendocrine tumors. JAMA Surg (2019) 154:134–40. doi: 10.1001/jamasurg.2018.3865 PMC6439661 30383112

[12] Sorbye H Baudin E Perren A. The problem of high-grade gastroenteropancreatic neuroendocrine neoplasms: Well-differentiated neuroendocrine tumors, neuroendocrine carcinomas, and beyond. Endocrinol Metab Clin North Am (2018) 47:683–98. doi: 10.1016/j.ecl.2018.05.001 30098724

[13] Kidd M Modlin I Öberg K. Towards a new classification of gastroenteropancreatic neuroendocrine neoplasms. Nat Rev Clin Oncol (2016) 13:691–705. doi: 10.1038/nrclinonc.2016.85 27273044

[14] Lv Y Han X Xu XF Ji Y Zhou YH Sun HC. Risk factors affecting prognosis in metachronous liver metastases from WHO classification G1 and G2 gastroenteropancreatic neuroendocrine tumors after initial R0 surgical resection. BMC Cancer (2019) 19:335. doi: 10.1186/s12885-019-5457-z 30961559 PMC6454726

[15] Toriyama K Yamamura T Nakamura M Maeda K Sawada T Mizutani Y. An evaluation of resectability among endoscopic treatment methods for rectal neuroendocrine tumors <10 mm. Arab J Gastroenterol (2021) 22:104–10. doi: 10.1016/j.ajg.2021.05.007 34053887

[16] Sivandzadeh GR Ejtehadi F Shoaee S Aminlari L Niknam R Taghavi AR. Endoscopic mucosal resection: still a reliable therapeutic option for gastrointestinal neuroendocrine tumors. BMC Gastroenterol (2021) 21:238. doi: 10.1186/s12876-021-01821-6 34030644 PMC8142474

[17] Wang L Shen J Zhang X Lu H Chu W. Retrospective analysis of the clinical effects of endoscopic mucosal dissection on treatment of early esophagogastric precancerous lesions. Clin Transl Oncol (2021) 23:731–7. doi: 10.1007/s12094-020-02462-z 32789667

[18] Banks M Uedo N Bhandari P Gotoda T. EMR achieves similar oncological outcomes as ESD for gastric neoplasia of <1cm, requiring less expertise, training and time. Gut. (2020) 69:1. doi: 10.1136/gutjnl-2019-319925 31586933

[19] Yazici C Boulay BR. Evolving role of the endoscopist in management of gastrointestinal neuroendocrine tumors. World J Gastroenterol (2017) 23:4847–55. doi: 10.3748/wjg.v23.i27.4847 PMC5526755 28785139

[20] Pagano N Ricci C Brighi N Ingaldi C Pugliese F Santini D. Incidental diagnosis of very small rectal neuroendocrine neoplasms: when should endoscopic submucosal dissection be performed? a single ENETS centre experience. Endocrine. (2019) 65:207–12. doi: 10.1007/s12020-019-01907-y 30919286

[21] Bertani E Ravizza D Milione M Massironi S Grana CM Zerini D. Neuroendocrine neoplasms of rectum: A management update. Cancer Treat Rev (2018) 66:45–55. doi: 10.1016/j.ctrv.2018.04.003 29684743

[22] Ramage JK De Herder WW Delle Fave G Ferolla P Ferone D Ito T. ENETS consensus guidelines update for colorectal neuroendocrine neoplasms. Neuroendocrinology. (2016) 103:139–43. doi: 10.1159/000443166 26730835

[23] Gu D Hu Y Ding H Wei J Chen K Liu H. CT radiomics may predict the grade of pancreatic neuroendocrine tumors: a multicenter study. Eur Radiol (2019) 29:6880–90. doi: 10.1007/s00330-019-06176-x 31227882

[24] Feng ST Luo Y Chan T Peng Z Chen J Chen M. CT evaluation of gastroenteric neuroendocrine tumors: Relationship between ct features and the pathologic classification. AJR Am J Roentgenol (2014) 203:W260–6. doi: 10.2214/AJR.13.11310 25148182

[25] Yu R Wachsman A. Imaging of neuroendocrine tumors: Indications, interpretations, limits, and pitfalls. Endocrinol Metab Clin North Am (2017) 46:795–814. doi: 10.1016/j.ecl.2017.04.008 28760239

[26] Hofland J Zandee WT de Herder WW. Role of biomarker tests for diagnosis of neuroendocrine tumours. Nat Rev Endocrinol (2018) 14:656–69. doi: 10.1038/s41574-018-0082-5 30158549

[27] O'Toole D Grossman A Gross D Delle Fave G Barkmanova J O'Connor J. ENETS consensus guidelines for the standards of care in neuroendocrine tumors: biochemical markers. Neuroendocrinology. (2009) 90:194–202. doi: 10.1159/000225948 19713711

[28] Collins GS Reitsma JB Altman DG Moons KG. Transparent reporting of a multivariable prediction model for individual prognosis or diagnosis (TRIPOD): the TRIPOD statement. BMJ (2015) 350:g7594. doi: 10.1161/CIRCULATIONAHA.114.014508 25569120

[29] Peduzzi P Concato J Kemper E Holford TR Feinstein AR. A simulation study of the number of events per variable in logistic regression analysis. J Clin Epidemiol (1996) 49:1373–9. doi: 10.1016/S0895-4356(96)00236-3 8970487

[30] Moons KG Kengne AP Woodward M Royston P Vergouwe Y Altman DG. Risk prediction models: I. development, internal validation, and assessing the incremental value of a new (bio)marker. Heart. (2012) 98:683–90. doi: 10.1136/heartjnl-2011-301246 22397945

[31] Balachandran VP Gonen M Smith JJ DeMatteo RP. Nomograms in oncology: more than meets the eye. Lancet Oncol (2015) 16:e173–80. doi: 10.1016/S1470-2045(14)71116-7 PMC4465353 25846097

[32] Toshima F Inoue D Komori T Yoshida K Yoneda N Minami T. Is the combination of MR and CT findings useful in determining the tumor grade of pancreatic neuroendocrine tumors? Jpn J Radiol (2017) 35:242–53. doi: 10.1007/s11604-017-0627-x 28258323

[33] Bian Y Li J Cao K Fang X Jiang H Ma C. Magnetic resonance imaging radiomic analysis can preoperatively predict G1 and G2/3 grades in patients with NF-pNETs. Abdom Radiol (NY) (2021) 46:667–80. doi: 10.1007/s00261-020-02706-0 32808056

[34] Ohki K Igarashi T Ashida H Takenaga S Shiraishi M Nozawa Y. Usefulness of texture analysis for grading pancreatic neuroendocrine tumors on contrast-enhanced computed tomography and apparent diffusion coefficient maps. Jpn J Radiol (2021) 39:66–75. doi: 10.1007/s11604-020-01038-9 32885378

[35] Liang W Yang P Huang R Xu L Wang J Liu W. A combined nomogram model to preoperatively predict histologic grade in pancreatic neuroendocrine tumors. Clin Cancer Res (2019) 25:584–94. doi: 10.1158/1078-0432.CCR-18-1305 30397175

[36] Bian Y Jiang H Ma C Wang L Zheng J Jin G. CT-based radiomics score for distinguishing between grade 1 and grade 2 nonfunctioning pancreatic neuroendocrine tumors. AJR Am J Roentgenol (2020) 215:852–63. doi: 10.2214/AJR.19.22123 32755201

[37] Chen X Wang Z. The differences between pancreatic neuroendocrine tumors grade 2 and grade 3-letter. Clin Cancer Res (2019) 25:4580. doi: 10.1158/1078-0432.CCR-19-0518 31308051

[38] Kim DW Kim HJ Kim KW Byun JH Song KB Kim JH. Neuroendocrine neoplasms of the pancreas at dynamic enhanced CT: Comparison between grade 3 neuroendocrine carcinoma and grade 1/2 neuroendocrine tumour. Eur Radiol (2015) 25:1375–83. doi: 10.1007/s00330-014-3532-z 25465713

[39] Guo C Zhuge X Wang Z Wang Q Sun K Feng Z. Textural analysis on contrast-enhanced CT in pancreatic neuroendocrine neoplasms: association with WHO grade. Abdom Radiol (NY) (2019) 44:576–85. doi: 10.1007/s00261-018-1763-1 30182253

[40] Guo C Chen X Xiao W Wang Q Sun K Wang Z. Pancreatic neuroendocrine neoplasms at magnetic resonance imaging: comparison between grade 3 and grade 1/2 tumors. Onco Targets Ther (2017) 10:1465–74. doi: 10.2147/OTT.S127803 PMC5349505 28331340

[41] Horiguchi S Kato H Shiraha H Tsutsumi K Yamamoto N Matsumoto K. Dynamic computed tomography is useful for prediction of pathological grade in pancreatic neuroendocrine neoplasm. J Gastroenterol Hepatol (2017) 32:925–31. doi: 10.1111/jgh.13594 27637470

[42] Wang Y Liang W. Limited clinical application of CT-based prediction model for pathologic grade of pancreatic neuroendocrine tumor. AJR Am J Roentgenol (2021) 216:W29. doi: 10.2214/AJR.20.25213 33729879

[43] Gut P Czarnywojtek A Sawicka-Gutaj N Woliński K Maciejewski A Komarnicki P. Determination of neuron-specific enolase in patients with midgut-type tumour treated with somatostatin analogues. Endokrynol Pol (2021) 72:308–18. doi: 10.5603/EP.a2021.0060 34292567

[44] Li Y Wu ZQ Xu Q Goyal H Xu HG. Development and validation of novel nomograms using serum tumor markers for the prediction of preoperative histologic grades in gastroenteropancreatic neuroendocrine tumors. Front Oncol (2021) 11:681149. doi: 10.3389/fonc.2021.681149 34109127 PMC8181758

[45] Mjønes P Sagatun L Nordrum IS Waldum HL. Neuron-specific enolase as an immunohistochemical marker is better than its reputation. J Histochem Cytochem (2017) 65:687–703. doi: 10.1369/0022155417733676 28972818 PMC5714096

[46] van Adrichem RC Kamp K Vandamme T Peeters M Feelders RA de Herder WW. Serum neuron-specific enolase level is an independent predictor of overall survival in patients with gastroenteropancreatic neuroendocrine tumors. Ann Oncol (2016) 27:746–7. doi: 10.1093/annonc/mdv626 26712902

[47] Sohda M Saeki H Kuwano H Miyazaki T Yokobori T Sano A. Diagnostic immunostaining and tumor markers predict the prognosis of esophageal neuroendocrine cell carcinoma patients. Ann Surg Oncol (2021) 28:7983–9. doi: 10.1245/s10434-021-09872-5 33843025

[48] Lv Y Han X Zhang C Fang Y Pu N Ji Y. Combined test of serum CgA and NSE improved the power of prognosis prediction of NF-pNETs. Endocr Connect (2018) 7:169–78. doi: 10.1530/EC-17-0276 PMC5776672 29191920

[49] de Klaver W Wisse PHA van Wifferen F Bosch LJW Jimenez CR van der Hulst RWM. Clinical validation of a multitarget fecal immunochemical test for colorectal cancer screening : A diagnostic test accuracy study. Ann Intern Med (2021) 174:1224–31. doi: 10.7326/M20-8270 34280333

[50] Bosch FTM Mulder FI Huisman MV Zwicker JI Di Nisio M Carrier M. Risk factors for gastrointestinal bleeding in patients with gastrointestinal cancer using edoxaban. J Thromb Haemost (2021) 19:3008–17. doi: 10.1111/jth.15516 PMC9292167 34455706

[51] Pan B Zhang W Chen W Zheng J Yang X Sun J. Establishment of the radiologic tumor invasion index based on radiomics splenic features and clinical factors to predict serous invasion of gastric cancer. Front Oncol (2021) 11:682456. doi: 10.3389/fonc.2021.682456 34434892 PMC8381151

[52] Zhai B Chen J Wu J Yang L Guo X Shao J. Predictive value of the hemoglobin, albumin, lymphocyte, and platelet (HALP) score and lymphocyte-to-monocyte ratio (LMR) in patients with non-small cell lung cancer after radical lung cancer surgery. Ann Transl Med (2021) 9:976. doi: 10.21037/atm-21-2120 34277776 PMC8267290

[53] Caro JJ Salas M Ward A Goss G. Anemia as an independent prognostic factor for survival in patients with cancer: a systemic, quantitative review. Cancer. (2001) 91:2214–21. doi: 10.1002/1097-0142(20010615)91:12<2214::AID-CNCR1251>3.0.CO;2-P 11413508

[54] Johanssen S Boivin M Lochs H Voderholzer W. The yield of wireless capsule endoscopy in the detection of neuroendocrine tumors in comparison with CT enteroclysis. Gastrointest Endosc (2006) 63:660–5. doi: 10.1016/j.gie.2005.11.055 16564869

[55] Delle Fave G O'Toole D Sundin A Taal B Ferolla P Ramage JK. ENETS consensus guidelines update for gastroduodenal neuroendocrine neoplasms. Neuroendocrinology. (2016) 103:119–24. doi: 10.1159/000443168 26784901

